# Randomized Trial on the Clinical Utility of a Novel Biomarker Panel to Identify Treatable Determinants of Chronic Pain

**DOI:** 10.3390/diagnostics10080513

**Published:** 2020-07-23

**Authors:** John Peabody, David Paculdo, Diana Tamondong-Lachica, Ian Theodore Cabaluna, Joshua Gunn

**Affiliations:** 1 College of Medicine, University of California, San Francisco, CA 94158, USA; 2 Fielding School of Public Health, University of California, Los Angeles, CA 90095, USA; 3 QURE Healthcare, San Francisco, CA 94133, USA; dpaculdo@qurehealthcare.com (D.P.); dlachica@qurehealthcare.com (D.T.-L.); icabaluna@qurehealthcare.com (I.T.C.); 4 Ethos Laboratories, Newport, KY 41071, USA; DrGunn@ETHOSRD.COM

**Keywords:** primary care, chronic pain, clinical utility, pain management, biomarker, oxidative stress, micronutrients, opioids, pain biomarker

## Abstract

Millions suffer daily from chronic pain diagnosed anatomically and treated with opioids. Research shows that underlying nutritional, metabolic and oxidative stressors, which drive the development or worsening of chronic pain, are not diagnosed despite the fact that treatment of these primary pain pathways relieves pain and increases function. One of the main reasons for this gap in care is the lack of a simple diagnostic assay to help clinicians make these diagnoses. We examined the clinical utility of a urine-based pain biomarker panel. Primary care physicians were randomized into the test group and compared to controls. We measured their ability to make the diagnosis and treat a total of nine standardized patients, with common but challenging cases of chronic pain, over two rounds of data collection in a pre–post design using a fixed-effects model. Intervention doctors received educational materials on a novel pain biomarker panel after the baseline round and had access to biomarker test results. Provider responses were measured against evidence-based criteria. The two study arms at baseline provided similar, poor care for three different primary pain pathways: nutritional deficiencies (5.0% control versus 9.2% intervention treated, *p* = 0.208), metabolic abnormalities (1.0% control versus 0% for intervention treated, *p* = 0.314), and oxidative stress (1.2% control versus 0% intervention treated, *p* = 0.152). After the introduction of the Foundation Pain Index (FPI) biomarker test, physicians in the intervention group were 41.5% more likely to make the diagnosis of a micronutrient deficiency, 29.4% more likely to identify a treatable metabolic abnormality and 26.1% more likely to identify an oxidative stressor. These diagnostic and treatment improvements were seen across all three case types, ranging from a relative +54% (*p* = 0.004) for chronic neuropathic pain to +35% (*p* = 0.007) in chronic pain from other causes to +38% (*p* = 0.002) in chronic pain with associated mental health issues. Intervention doctors were also 75.1% more likely to provide a non-opioid treatment to patients on chronic opioids (O.R. 1.8, 95% C.I. 0.8–3.7), 62% less likely to order unnecessary imaging for their patients with low back pain (O.R. 0.38, 95% C.I. 0.15–0.97) and 66% less likely to order an unnecessary pain referral (O.R. 0.34, 95% C.I. 0.13–0.90). This experimental study showed significant clinical utility of a validated pain biomarker panel that determines nutritional deficiencies, metabolic abnormalities and oxidative stressors that drive underlying treatable causes of pain. When integrated into routine primary care practice, this testing approach could considerably improve diagnostic accuracy and provide more targeted, non-opioid treatments for patients suffering from chronic pain.

## 1. Introduction

The chronic pain burden in the United States has enormous physical, emotional, social and economic consequences [1]. Estimates are that 13% (25 million) adults suffer from low back pain [2], 3 to 7 million have chronic migraine [3], and 10 to 20% of patients with diabetes (6 to 8 million) suffer from painful diabetic or idiopathic polyneuropathy [4,5]. In 2018, more than 1.5 million long-term opioid prescriptions were filled every week [6]. These manifestations affect the daily function of more than 20 million Americans [7]. Chronic pain was also associated with higher rates of consultation and perceived unfavorable general health [8].

Effective treatment of chronic pain has been elusive. In a recent publication, we reported that the overall care for patients with chronic pain syndromes was highly varied and often inadequate [9]. The overall diagnostic accuracy, which we defined as the identification of the primary pain diagnoses, the primary pain pathway and the secondary co-morbid diagnoses, was only 59.6% (S.D. 27.6%). One of the important findings of this study was that diagnosing the primary pain pathway was routinely missed and, with that, not treated. Specifically, providers rarely or never treated underlying biochemical aberrations including nutritional deficiencies (5.2%), oxidative stress (0.7%), or metabolic abnormalities (0%). This is surprising considering the rapidly growing body of high-quality randomized controlled trial data that provide conclusive evidence that treatment with vitamin B (B6, B9, B12), acetyl-L-carnitine, alpha-lipoic acid, coenzyme Q10 (CoQ10), and *N*-acetylcysteine mitigates severe and persistent pain while improving function [10,11,12,13,14].

A key takeaway is that testing, which is necessary to make the diagnosis and initiate treatment, is not being performed. Even when indicated, we found that necessary tests, such as a chemistry panel, testing for thyroid function (30%), fasting blood sugar (23%) or vitamin B12/B6 levels (22%) were performed less than half the time. Tests identifying oxidative stress such as carnitine, CoQ10 and glutathione assays and nerve health markers such as 3-hydroxypropyl mercapturic acid (3-HPMA) were simply not available or considered for non-pain diagnoses, which, if done, would have uncovered underlying biology of biochemical pain pathways. Our understanding of the biology of chronic pain syndromes is categorized into the three main contributors: micronutrient deficiencies, metabolic abnormalities and oxidative stress [15,16,17]. More individualized diagnosis and more targeted care addressing these co-factors, most pain experts agree, would be an important advance in chronic pain care [18].

For the clinician already struggling to relieve the suffering of their chronic pain patients, other recent research has brought the diagnostic and therapeutic shortcomings we have uncovered into stark relief. In a meta-analysis, nutrition-based interventions (either through alteration of overall diet or supplementation of single nutrient) were identified that could have reduced the severity and intensity of pain [19]. We know too that reduced levels of vitamin B12, for example, lead to nerve damage and degeneration of the spinal cord, while low vitamin B6 levels present as neuropathy with numbness, paresthesia or burning pain starting from the feet and up to the legs and arms [20]. Another study identified specific nutritional deficiencies in vitamins B12, B6, and D; in CoQ10 and glutathione; and in mineral deficiencies of zinc and selenium as contributing factors to chronic pain. Importantly, treating these deficiencies improves the patients’ chronic pain [20,21,22]. The literature is clear, too, that other nutritional deficiencies will arise from the treatment of concomitant diseases—chronic kidney disease, diabetes, and gastroesophageal reflux disease (GERD)—that can cause vitamin deficiencies associated with chronic pain, e.g., hemodialysis lowers serum vitamin B6 levels while metformin and proton pump inhibitors reduce vitamin B12 absorption in the gut [23,24].

Nearly all degenerative diseases appear to have an underlying diet-induced, pro-inflammatory state that can be mitigated if diagnosed [25,26]. Essential fatty acid imbalance and deficiency in antioxidants due to inadequate intake of fruits and vegetables, chronic tobacco and alcohol use are also believed to contribute to a chronic inflammatory state. Studies have shown that elevated homocysteine levels, for example, cause inflammation by increasing arachidonic acid and the pro-inflammatory prostaglandin E2 production [20,27]. Increased homocysteine levels indicate that there is an intimate relationship between inflammation and chronic pain [27].

Metabolic abnormalities contribute to nerve health impairment and are also largely overlooked. Free radicals are not only believed to produce pain, they also lower the local nociceptive threshold, leading to hyperalgesia [28,29,30]. Culprits include low levels of glutathione, CoQ10, carnitine and vitamin B12. Acetaminophen, commonly used in pain control, for instance, can drastically lower glutathione levels in the body, manifested by elevated pyroglutamate in the urine. Low CoQ10 levels, indicated by an excess of hydroxymethylglutarate, results in mitochondrial abnormalities such as elevated lactate and citric acid intermediates. This is clinically manifested by fatigue, muscle weakness, pain and peripheral neuropathy and is the mechanism behind statin-induced myopathy [31]. Carnitine deficiency results from a wide range of conditions such as diabetes, sepsis, cardiomyopathy, malnutrition, cirrhosis, endocrine disorders and aging, and it has been implicated in causing painful neuropathies. Elevated urine ethylmalonate levels can also be used to diagnose carnitine deficiency, apart from measuring free and total carnitine levels in the blood.

Using validated biomarkers—to detect nutritional, inflammatory and oxidative stress pathologies, and other nerve/metabolic abnormalities that are directly correlated with presence and severity of chronic pain—holds promise if they are able to identify treatable causes of chronic pain. Successful treatment based upon mechanistic biomarkers of pain could obviate the need for long-term, potentially hazardous use of opioids and provide specific treatments for the cause of pain [20]. The addition of a reliable and more convenient method of biomarker testing, such as urine assays, however, is needed if clinicians are going to improve the diagnosis and better personalize the treatment for chronic pain patients.

We conducted a randomized control trial among primary care practitioners caring for patients with chronic pain. Our hypotheses were that doctors who had a newly available, easy to use, and convenient diagnostic test panel that detects nutritional deficiencies, metabolic abnormalities and oxidative stressors—referred to collectively herein as the primary pain pathways—would more ably diagnose and treat patients with chronic pain.

## 2. Materials and Methods

The Functional Inflammatory and Nutritional Diagnostic Utility Standard—QURE Biomarker (FIND-US QB) study is a randomized controlled trial conducted between March and May 2020 to measure the diagnostic accuracy of identifying and managing primary pain pathways caused by nutritional deficiencies, metabolic abnormalities and oxidative stress and to determine whether this information led to better management (i.e., treatment) of chronic pain. We measured clinical performance in nine different simulated patients with common pain diagnoses and we determined the clinical utility of the Functional Biomarkers of Pain (FBP) panel and Foundation Pain Index (FPI) score among primary care physicians (PCPs) and pain specialists practicing in the United States.

### 2.1. Ethics

The study was conducted in accordance with ethical standards and approved by the Advarra Institutional Review Board, Columbia, MD (01-ETH-2020, 21 January 2020). The study was also listed in clinicaltrials.gov (NCT04266821, 12 February 2020). All participants provided online informed consent to be in the study.

### 2.2. Physician Selection

We recruited 238 participants from a national list of PCPs and pain specialists gathered from multiple sources, including rosters from relevant conferences, medical associations, and professional organizations.

A physician was deemed eligible if they met the following criteria: (1) board-certified physician for at least 2 years, (2) average at least 20 h per week of clinical and patient care duties over the last 6 months, (3) at least 15% of patient panel with chronic pain, (4) prescribe opioids as part of their regular practice, (5) practice in the United States, (6) English speaking, (7) access to the internet, and (8) voluntarily consented. Of the 238 recruited physicians, 20 did not complete the eligibility survey and 45 providers did not meet the requirements of the study, resulting in 173 eligible physician participants. Of the 173 participants who began the study, 19 did not complete the first round of data collection, and 3 did not complete the second round of data collection, leaving 151 providers whose data were used in the FIND-US QB study. We found no significant difference in physician or practice demographics between the 22 who did not complete the study and the 151 who did (*p* > 0.05 for all characteristics).

All participants completed a health provider questionnaire as part of the data collection. The questionnaire consisted of questions on provider demographics, training and their practice environment.

### 2.3. Intervention

Participants were randomly assigned to either intervention or control group in a 1:1 ratio using a simple coin flip methodology. The intervention group received educational materials on the Functional Biomarkers of Pain test/Foundation Pain Index score after completing the first round of data collection. These IRB-approved materials are: a slide deck with overview and clinical utility of the FPI test, a 2-pager that included frequently asked questions (FAQs) about FPI, and a sample FPI report. These have been designed to replicate the actual promotional materials to introduce the FBP panel/FPI score to physicians.

The FBP panel is a multi-analyte assay that determines the urine levels of eleven endogenous pain biomarkers that were selected based on the high prevalence of abnormalities among chronic pain patients and potential for safe, targeted treatment. The included markers are those related to micronutrient status (methylmalonic acid, xanthurenate, homocysteine), cytokine-mediated inflammation (quinolinic acid, kynurenate), oxidative stress (pyroglutamic acid, hydroxymethylglutarate, ethylmalonate), and neurotransmitter synthesis and metabolism (5-hydroxyindoleacetic acid and vanilmandelate) (Table S1). Results of the FBP are summarized and presented as a (FPI) score, which is a number scaled between 0 and 100 and generated by a validated algorithm to indicate the likelihood that detected biomarker abnormalities are contributing to the patient’s pain.

Measurement Using Clinical Performance and Value (CPV^®^) vignettes. CPVs are online patient simulations that have been validated in standardized patients and are used to measure clinical care [32,33]. The vignettes use open-ended questions that allow physicians to (1) request and review patient histories, (2) make a physical examination of the patient, and (3) order diagnostic tests and procedures to recreate an actual patient visit. Once participants complete these domains of care, they are then charged with (4) making a diagnosis with a treatment plan and follow up. These have been used to evaluate and compare clinical practices of health care providers in many countries, covering a wide range of diseases [34,35,36]. A 3–5% improvement in CPV scores has been shown to reflect actual improvement in real patients [37]. A team of physicians designed the cases to resemble a typical patient presenting with chronic pain visiting their PCP.

Scorers—blinded to the participant’s study arm and identity—used explicit, pre-determined evidence-based criteria to measure physician care. An overall score and a care score are then generated in three specific clinical domains: ordering diagnostic workup, making the diagnosis, and developing and outlining a treatment plan.

The CPV cases were designed to reflect the complexity of chronic pain as influenced by psychosocial, clinical, and biological factors that can be the bases for curative interventions. We were particularly interested in whether the study participants could detect (i.e., could diagnose) the primary pain pathways caused by nutritional deficiencies, metabolic abnormalities and oxidative stress and whether this information led to management (i.e., treatment) based upon the mechanistic biomarkers of pain. Accordingly, diagnostic accuracy was determined several ways: by the primary pain diagnosis, by diagnosing the primary pain pathways and by diagnosing any co-morbid diagnosis. Primary pain diagnoses indicate the pain syndromes and their respective anatomic and pathologic categories, for example, lumbar spinal stenosis and distal symmetric polyneuropathy.

Secondary diagnoses are comorbidities such as mental health disorders, diabetes and chronic kidney disease that may contribute to pain severity and duration. The diagnosis of the primary pain biomarkers is distinguished from the primary pain diagnoses because these represent the underlying, potentially reversible contributors to pain, such as carnitine, CoQ10 and glutathione deficiencies which drive oxidative stress or vitamin B12 and B6 deficiencies which compromise nerve health and function [20].

Three case types representing common but difficult-to-control chronic pain syndromes were used (Table 1). In all, there were nine CPV cases, with three in each group: (1) chronic central pain with comorbidities of depression or anxiety, (2) chronic neuropathic pain, and (3) chronic pain associated with fibromyalgia, chronic fatigue and spinal stenosis. Each physician completed six total cases (one from each group): three in each of two rounds of data collection.

All participants were randomly assigned to complete three cases for the first or baseline round. During the second or post intervention round, participants were given three additional CPV cases, but only the intervention group had access to simulated FPI test results.

### 2.4. Analysis

The primary outcome was the change in the diagnostic and treatment scores. The scores were based upon pre-determined, evidence-based, quality of care criteria and reported on a percentage basis. Other research indicates that an absolute improvement of more than 3% results in a change in real-world clinical practice [35]. We looked to determine whether the introduction of the FBP test and the accompanying FPI score improved the intervention groups ability to diagnose and treat the primary pain pathways (nutritional deficiencies, metabolic abnormalities and oxidative stress markers) to the patient’s pain. Subanalyses included care and treatment of patients on chronic opioids and use of imaging studies in patients with low back pain.

Summary statistics were determined for all variables. Numerical variables were summarized through mean and standard deviation. For categorical outcomes, we used the chi-squared test and logistic regression for multivariate modeling. For continuous outcomes, *t*-test and linear regression were performed. CPV domain and treatment scores were adjusted using multiple linear regression model to control potential confounders. All analyses were performed in Stata 14.2.

## 3. Results

A total of 151 providers participated in the study; 75 were randomized to the intervention arm and 76 were randomized to the control group. We found no difference in baseline characteristics between the two study arms (*p* > 0.05 for all) (Table 2).

The 151 PCPs saw a total of 906 CPV patients (one from each case type over two rounds of data collection), with an even split between the three case types: (1) chronic central pain with mental health-related comorbidities, (2) chronic neuropathic pain, and (3) chronic pain associated with other causes. Within each case type, patients had treatable pain pathway deficiencies due to underlying nutritional deficiencies, metabolic abnormalities and oxidative stress.

We compared the PCPs on their diagnostic accuracy—half with and half without FPI testing—to evaluate how well they determined the primary causes of pain; the underlying nutritional, metabolic and oxidative markers contributing to the chronic pain; and the secondary diagnoses. We then measured the rates of appropriate treatment for the chronic pain and treatment of any underlying pain pathway. We performed subanalyses on the patients treated with opioids (five of the nine cases) and use of imaging studies for patients with chronic low back pain (four cases).

At baseline, when providers did not have FPI testing available to them, we found no difference in patient care between the two study arms (Table 3). There was no difference in the way they diagnosed or treated these patients (23.6% + 15.0% for control vs. 21.0% + 13.4% for intervention, *p* = 0.055). While both arms were equally adept at identifying the correct primary cause of the chronic pain (83.2% for controls vs. 79.0% for intervention, *p* = 0.259), nearly everyone in both groups did not identify the underlying primary pain pathway whether it was the nutritional deficiencies that caused the pain (7.1% for controls vs. 8.9% for intervention, *p* = 0.647) or the metabolic and oxidative stressors (1.0% for controls vs. 0% for intervention, *p* = 0.157). The two groups were also statistically indistinguishable in how they treated these patients at baseline. In the treatment domain, the overall treatment scores in both groups were 15.9% + 17.3% for control versus 14.6% + 14.4% (*p* = 0.387). The two study arms at baseline provided similar poor treatment for the three different underlying pain pathways: nutritional deficiencies (5.0% control versus 9.2% intervention, *p* = 0.208), metabolic abnormalities (1.0% control versus 0% for intervention, *p* = 0.314), and oxidative stressors (1.2% control versus 0% intervention, *p* = 0.152).

### 3.1. Clinical Performance after the Introduction of the Foundation Pain Index (FPI) Test

In the second round of data collection, after the introduction of the FPI pain biomarker test, physicians in the intervention group were 9.3% more likely to make a more accurate aggregate diagnosis and provide better overall treatment (*p* < 0.001) (Table 4a), a relative improvement of ~40%. These absolute improvements were seen across all three case types, ranging from +8.6% (*p* = 0.004, +54% relative) in case type 2 (chronic neuropathy) to +8.9% (*p* = 0.007, +35% relative) in case type 3 (chronic pain from other causes) to +10.3% (*p* = 0.002, +38% relative) in case type 1 (chronic pain with associated mental health issues). Below we examine where these overall improvements occurred, starting with primary and secondary diagnosis treatments and then looking closely at the primary pain pathways.

### 3.2. Diagnosing Chronic Pain and Co-Morbidities

The intervention and control group made the primary pain diagnosis—whether it was low back pain, phantom limb pain, neuropathy, migraine, medication overuse headache or chronic fatigue syndrome—equally well in the second round, averaging 79.0% and 83.2%, respectively, with no statistically significant improvement compared to baseline (*p* = 0.866 for controls and *p* = 0.080 for intervention) (Table 3). Similarly, we found no statistically significant improvement in the secondary, comorbid diagnostic accuracy of approximately 40% in either round for anxiety/depression, diabetes, dyslipidemia, chronic kidney disease and GERD for either the intervention group (*p* = 0.504) or the control group (*p* = 0.701).

### 3.3. Treatment of Chronic Pain and Co-Morbidities

We found no difference between intervention and control groups’ treatment of chronic pain in round 2 (*p* = 0.432), with both arms decreasing slightly round over round by approximately 10%. In a pattern we would see throughout our analyses, those who made the primary diagnosis correctly were 77% (95% C.I. 25–151%) more likely to correctly treat their patient’s pain (*p* = 0.001). We also found no significant difference in the treatment of secondary diagnoses (e.g., GERD, diabetes, and anxiety/depression) between study arms (*p* = 0.347 for round 1 and *p* = 0.576 for round 2), and again both arms decreased significantly from baseline to round 2 (*p* = 0.020 for intervention and *p* = 0.007 for control). We once more observed that those correctly making the secondary diagnosis gave the correct treatment 63.8% of the time compared to 30.6% of the time for those who did not. These findings were robust in our regression analysis: those who made the correct secondary diagnosis were 322% (210–576%) more likely to treat the co-morbid condition than those who did not.

### 3.4. Diagnosing Pain Pathways

However, when we looked more closely at the diagnostic identification of the primary pain pathways, we saw a very different picture. Among the cases with micronutrient deficiency, after the FPI test was introduced, the intervention group recognized the nutritional deficiencies 41.5% of the time in round 2 compared to only 13.1% in the control group and a 32.6% improvement over baseline (*p* < 0.001). These results were further substantiated in the multivariate regression, wherein intervention physicians were 4.1 times more likely to identify the pain pathway (95% C.I. 1.1–14.4) compared to controls (Table 4b). We saw a similar large increase in detecting metabolic abnormalities, with intervention physicians making the diagnosis in 29.4% of cases in round 2 versus 0% in this group at baseline, compared to 1.1% of controls (*p* < 0.001), which showed no improvement at all from baseline (*p* = 0.929). Similarly, we found an increase in identifying oxidative stress for the intervention physicians who detected it in 26.1% of cases versus 0.5% for controls in round 2 (*p* < 0.001) and 0% at baseline (*p* < 0.001).

### 3.5. Treating Pain Pathways

We next investigated how well physicians did at treating the primary pain pathways. The treatment of nutritional deficiency among the intervention doctors was 59.5%, dramatically better compared to 8.8% for control doctors in round 2 (*p* < 0.001) and compared to a 9.2% treatment rate for the intervention doctors at baseline (*p* < 0.001). In our regression model, those who made the correct diagnosis were 61.2 times more likely to treat the nutritional deficiency than those who did not (95% C.I. 24.0–156.1; Table 4c).

The intervention doctors were also more likely to treat oxidative stress (carnitine, CoQ10 and glutathione depletion) in their patients. In round 2, those using the FPI results treated the identified oxidative stress 26.1% of the time compared to control doctors in round 2 (0.5%), which was also the treatment rates for these patients at baseline (0%) (*p* < 0.001 for both). The regression analysis again confirmed that those who made the diagnosis of oxidative stress were significantly more likely to treat the oxidative stress (O.R. 5.9, 95% C.I. 2.4–14.5) and this happened more often among doctors over the age of 50 who provided treatment for oxidative stress ten times more often than younger doctors (O.R. 10.5, 95% C.I. 1.3–82.1).

Our findings, demonstrating the utility of the FPI test, were also seen in treating metabolic abnormalities that affect nerve and neurotransmitter status (e.g., 5-HTP for low 5-HIAA, N-acetyl-L-cysteine and alpha lipoic acid for high 3-HPMA). In round 2, the intervention doctors treated these conditions 29.4% of the time, compared to 1.1% for control doctors and 0.5% for intervention and control doctors in round 1 (*p* < 0.001 for both). Making the diagnosis of metabolic abnormalities was highly predictive for treatment (O.R. 6.3, 95% C.I. 1.6–25.2), with no other variables approaching significance.

### 3.6. Patients with Mental Health Diagnoses

Among the secondary diagnoses, we examined those patients who had a co-morbid mental health condition and determined whether FPI testing had any impact on diagnosing or treating depression or anxiety. Diagnosis of mental health illness, however, did not change between study arms in round 2 (48.0% for controls versus 48.9% for intervention, *p* = 0.900 within round 2), and there was no significant improvement in diagnosis between rounds for the intervention group (*p* = 0.185) or the control group (*p* = 0.296). Treatment again was linked to making the correct diagnosis (O.R. 7.9, 95% C.I. 4.5–13.7).

### 3.7. Patients on Chronic Opioids

Among patients who were on long-term opioids, the intervention group was no more likely to make the correct diagnosis (86.9% versus 81.9%, *p* = 0.266) after the introduction of the FPI test compared to controls. However, intervention doctors improved significantly over their baseline (77.0%, *p* = 0.038) while controls did not (82.5%, *p* = 0.829).

This ability to identify the primary pain pathway diagnosis led to better treatment. The intervention doctors treated their opioid patients for nutritional deficiencies 33.9% of the time versus 4.7% for controls in the second round (*p* < 0.001) and compared to 1.2% at baseline for both groups (*p* < 0.001). The treatment of oxidative stress also increased to 21.0% for intervention doctors compared to 0% in the control arm and compared to 0% at baseline for both (*p* < 0.001). Treating metabolic abnormalities followed a similar pattern, with intervention doctors improving their treatment of the opioid patients significantly (0% in round 1 to 23.6% in round 2, *p* < 0.001) and control doctors not showing any improvements in round 2 (0%, *p* < 0.001 compared to intervention).

We did not find that either group tapered their patients off opioids more often in the second round. When indicated, both sets of participants gradually reduced the opioid doses in 22.0% of the cases across both rounds and both groups (*p* = 0.477). Intervention doctors, however, were 75.1% more likely to provide a non-opioid treatment to their patients (O.R. 1.8, 95% C.I. 0.8–3.7) (Table 4d).

### 3.8. Unnecessary Imaging and Pain Referrals for Low Back Pain

We examined the use of unnecessary imaging (MRI, CT, and X-ray) and referrals for pain management in patients who had low back pain. Unnecessary imaging decreased in the intervention arm to 35.3% of the cases compared to 58.5% in the control group in round 2 (*p* = 0.005) and compared to the combined baseline unnecessary imaging of 52.3% (*p* = 0.012). A multivariate logistic model accounting for provider and practice characteristics showed that intervention providers were 62% less likely to order unnecessary imaging (O.R. 0.38, 95% C.I. 0.15–0.97). Intervention providers were also 66% less likely to order an unnecessary pain referral for their patients with low back pain (O.R. 0.34, 95% C.I. 0.13–0.90).

## 4. Discussion

Our understanding of how nutritional deficiencies, metabolic abnormalities and oxidative stress exacerbate chronic pain has grown in recent years. A growing body of literature indicates the value of targeted interventions aimed at identifying and addressing abnormal biomarker findings for the management and mitigation of various primary pain syndromes [22,38,39,40]. Collectively, diagnosing and treating these pain pathways hold exciting promise in a clinical field filled with frustration for physicians and especially patients. This enthusiasm has led to calls for more high-quality studies to understand and improve the efficiency in detecting and treating pain pathways [41,42].

The FIND-US QB study introduced the Foundation Pain Index (FPI), a urine-based assay, that detects an extensive array of pain biomarkers in an easily interpretable and actionable numeric score into primary care practices. We know from our previous research that pain pathways are rarely explored, diagnosed, and much less treated in these settings [9]. This led us to hypothesize that accurate diagnosis and treatment are hampered, fundamentally, because comprehensive testing is not available. We recruited a nationally representative sample of 151 mostly primary care providers to test whether this assay improved the diagnosis and treatment among nine common patient presentations of chronic pain. We used simulated patients—a validated and widely used method to determine clinical utility and clinical value—to control for patient-level variability and make it possible to isolate the effects of a new test on clinical decision making. In all, we studied the testing effects on a total of 906 simulated patients presenting with common pain syndromes and co-morbidities analyzed in a pre–post randomized controlled design where half the physicians had use of the test while the other half did not.

We observed significant improvement in diagnostic accuracy and the quality of treatment across every pain syndrome after the introduction of the FPI test. We divided our analysis of the pain pathways into three categories. Nutritional deficiencies, which are perhaps the most familiar to physicians were treated seven times more often compared to controls. There was a similar story in the treatment of oxidative stress, where treatment increased from 0% to 26.1% for the intervention group, over 50 times higher in round 2 compared to the control group. Likewise, the metabolic abnormalities, which are often iatrogenic (e.g., prolonged use of acetaminophen that depletes glutathione, metformin and PPI for B12 deficiency), were 27 times more likely to be recognized and managed after introduction of the FPI score.

Despite these dramatic improvements, primary pain pathways remain underdiagnosed and undertreated. At baseline, pain pathways were diagnosed and treated only 4.5% and 5.3% of the time across both groups, respectively. This finding is consistent with other studies on primary care practice in the literature [43]. With the introduction of FPI testing, diagnostic accuracy increased to 37.4%, and successful diagnosis led to therapeutic intervention in 85.7% of cases. Underdiagnosis was not confined to the primary pain pathways. The secondary comorbidities were identified correctly 40% of the time and even the primary diagnosis was made correctly only 80% of the time. This study and others like it confirm that diagnostic errors and omissions consistently lead to inadequate treatment [34,35,44]. Conversely, when the diagnosis was made, the correct treatment ensued 77% of the time for the primary diagnosis and 32% for the secondary diagnoses. In our subanalyses of depression and anxiety, both well known to be closely associated to chronic pain, providers were eight times more likely to treat if these are diagnosed correctly.

The introduction of the FPI test also indicates that there is a substantial opportunity to improve care among patients on long-term opioid therapy. Intervention doctors were more likely to identify treatable causes of chronic pain (33% for nutritional deficiency, 21% for oxidative stress and 24% for metabolic abnormalities) and were more likely to provide targeted interventions (based on the test) as well as non-opioid therapies, compared to their control counterparts. We did not find that identifying and treating the primary pain pathways reduced opioid use, perhaps because we were underpowered to make this determination. Future studies using longitudinal data and patient-reported data will be needed to determine whether patients receiving definitive treatment for their primary pain pathways could result in a decreased need for opioids.

Unless we are considering serious diseases such as cancer or systemic infection, most musculoskeletal regional pain such as low back pain does not require diagnostic imaging [8]. We found that FPI testing reduced unnecessary imaging and unneeded pain clinic referrals by 62% and 66%, respectively. This finding directly supports the intuition that better diagnoses and treatment will lead to lower utilization costs that might include other care elements we did not test for such as visits to the PC doctor and prescription costs, which is something future research might pursue.

Limitations. While our study demonstrates that pain pathway biomarker testing can significantly influence clinical practice towards more targeted and less opioid-reliant treatments, there is still ample head room to personalize pain management and individualize care. CPVs are validated to measure clinical practice and are a novel way to demonstrate clinical utility but patient-level data on subjective pain outcomes would be helpful to further elucidate the benefits of FPI testing. Additional studies, including studies that leverage this sample frame, are planned to compare our diagnostic findings and to look at patient-level impact. While there is currently an emphasis on multidisciplinary non-pharmacologic therapies for chronic pain, we did not address how or whether these should be integrated with biomarker testing. This study also did not consider the practice impact opportunities for the provider and patient satisfaction. It is highly likely that more targeted therapies with medicinal treatments other than analgesics would be welcome. Lastly, we only looked at three pain pathways. Future studies will need to look at other pathways such as neurohormonal regulation, neuroimaging-based brain biomarkers and those determined by genetic aberrations related to pain perception. Furthermore, we only looked at a select set of common clinical conditions and did not investigate other possible conditions, e.g., arthritides, visceral pain, or earlier biomarker testing for patients yet to receive opioid prescriptions or other interventions.

Even with the dramatic improvements seen with the introduction of a urine-based test, we did not address the overall reason why awareness of the primary pain pathways is so low. We believe that this study strongly indicates that increased awareness comes with the availability of a simple urine-based assay that leads to improved diagnosis and treatment by providing objective data on nutritional, metabolic and oxidative stressors that, if treated, mitigate chronic pain.

## 5. Conclusions

Using a randomized experimental design, this nationally representative study of primary care providers showed marked clinical utility of the FBP test/FPI score. We found that this novel comprehensive pain biomarker assay increased diagnostic accuracy dramatically for the primary pain pathways and, when diagnosed, led to significantly greater and more targeted treatment in patients with chronic pain.

## Figures and Tables

**Table 1:** List of Diagnoses by Clinical Performance and Value (CPV) Case Type.

Case	Primary Diagnosis	Primary Contributing Diagnosis	Secondary Diagnosis/Co-Morbidity
**Case Type 1: Chronic Central Pain with Mental Health-Related Comorbidities**			
1A	Lumbar spinal stenosis	Vitamin B12 deficiency Low serotonin synthesis	Depression Hypertension
1B	Phantom limb pain	Vitamin B12 and B6 deficiencies	Anxiety disorder Hypertension Dyslipidemia
1C	Non-specific chronic low back pain	-	Depression
**Case Type 2: Chronic Neuropathic Pain**			
2A	Distal symmetric polyneuropathy, likely from diabetes	Vitamin B12 deficiency Carnitine depletion	Type 2 DM CKD stage 3 Dyslipidemia
2B	Distal symmetric polyneuropathy, idiopathic	Functional vitamin B12 deficiency Carnitine depletion	Type 1 DM CKD stage 2 GERD
2C	Distal symmetric polyneuropathy, idiopathic	-	Depression Obesity
**Case Type 3: Chronic Pain Associated with Other Causes**			
3A	Intractable migraine Medication overuse headache	Glutathione depletion from chronic acetaminophen intake Acrolein exposure	Fibromyalgia
3B	Chronic pain syndrome Chronic fatigue	Severe vitamin B6 deficiency Acrolein exposure	-
3C	Lumbar spinal stenosis	-	GERD Opioid-related constipation and sedation

**Table 2:** Comparison of Intervention and Control Provider Characteristics.

Variables	Control	Intervention	*p*-Value
*N*	76	75	--
Male	72.4%	80.0%	0.271
Age	55.4 ± 9.1	55.9 ± 8.7	0.707
Board Certification *			
Family Medicine	52.6%	50.7%	0.809
Internal Medicine	44.7%	48.0%	0.688
Neurology	0.0%	1.3%	0.312
PM&R	2.6%	2.7%	0.989
Other	4.0%	9.3%	0.183
Years in Practice	25.3 ± 8.4	27.3 ± 9.0	0.151
**Region**			
Midwest	25.0%	16.0%	0.434
Northeast	21.1%	29.3%	
South	19.7%	17.3%	
West	34.2%	37.3%	
**Locale**			
Urban	29.0%	26.7%	0.791
Suburban	60.5%	65.3%	
Rural	10.5%	8.0%	
Employed by Practice, %	73.7%	85.3%	0.076
**Practice Setting**			
Private Practice, Solo	18.4%	18.7%	0.614
Private Practice, Single Specialty	31.6%	42.7%	
Private Practice, Multi-Specialty	35.5%	25.3%	
Hospital-Based	9.2%	9.3%	
Federally-Qualified Health Center	5.3%	4.0%	
Outpatient Time, %	89.0% ± 24.1%	91.2% ± 18.5%	0.526
**Payer Type**			
Public (Medicare/Medicaid)	42.7% ± 19.7%	40.3% ± 18.5%	0.444
Commercial	48.1% ± 21.7%	50.9% ± 20.7%	0.434
Self	5.4% ± 6.0%	6.8% ± 13.0%	0.371
Other	3.8% ± 16.1%	2.0% ± 4.5%	0.356
**CMS Quality Participation ^1^**			
MIPS	29.0%	40.0%	0.153
BPCI	10.5%	13.3%	0.595
Other	4.0%	6.7%	0.456
Do not participate	43.4%	32.0%	0.148
Don’t know	21.1%	12.0%	0.135
Receive Quality Bonus	44.7%	45.3%	0.941

* Does not sum to 100% because providers could choose more than one option.

**Table 3:** Comparison of Control and Intervention Performance across Two Rounds of Data Collection.

Diagnosis and Treatment	Round		
	1	2	*p*-value
Control	23.6% ± 15.0%	20.1% ± 11.4%	0.006 *
Intervention	21.0% ± 13.4%	26.8% ± 16.9%	<0.001 *
*p*-value	0.055 *	<0.001 *	<0.001 ^†^
**Primary Pain Diagnosis**	**Round**		
	1	2	*p*-value
Control	83.2%	83.8%	0.866 ^‡^
Intervention	79.0%	85.3%	0.080 ^‡^
*p*-value	0.259 ^‡^	0.646 ^‡^	0.269 ^§^
**Secondary Diagnosis**	**Round**		
	1	2	*p*-value
Control	41.0%	39.1%	0.701 ^‡^
Intervention	44.3%	41.0%	0.504 ^‡^
*p*-value	0.498 ^‡^	0.699 ^‡^	0.839 ^§^
**Nutritional Deficiency Diagnosis**	**Round**		
	1	2	*p*-value
Control	7.1%	13.1%	0.164 ^‡^
Intervention	8.9%	41.5%	<0.001 ^‡^
*p*-value	0.647 ^‡^	<0.001 ^‡^	<0.001 ^§^
**Treatment of Nutritional Deficiency**	**Round**		
	1	2	*p*-value
Control	5.0%	8.8%	0.249 ^‡^
Intervention	9.2%	59.5%	<0.001 ^‡^
*p*-value	0.208^‡^	<0.001 ^‡^	0.001 ^§^
**Treatment of Oxidative Stress**	**Round**		
	1	2	*p*-value
Control	1.2%	0.5%	0.494 ^‡^
Intervention	0.0%	26.1%	<0.001 ^‡^
*p*-value	0.152 ^‡^	<0.001 ^‡^	<0.001 ^§^
**Treatment of Metabolic Abnormality**	**Round**		
	1	2	*p*-value
Control	1.0%	1.1%	0.929 ^‡^
Intervention	0.0%	29.4%	<0.001 ^‡^
*p*-value	0.314 ^‡^	<0.001 ^‡^	<0.001 ^§^

* Student’s *t*-test; † linear regression model; ‡ chi-squared test; § logistic regression model.

**Table:** 

(a)		
Variable	Coef.	*p* > t
**Male**	−2.7	0.017
**Age group (ref age < 50)**		
50–59	3.1	0.009
60+	5.5	0.000
**Family medicine**	1.6	0.100
**Northeast**	−2.1	0.060
**Urban setting**	0.0	0.980
**Solo, private practice**	−3.4	0.007
**Intervention**	−2.5	0.065
**Round**	−3.5	0.008
Intervention * round	9.3	0.000
**Constant**	23.0	0.000

**Table:** 

(b)			
		[95% Conf. Interval]	
Variable	Odds Ratio	Lower	Upper
**Male**	0.5	0.3	1.0
**Age group (ref age < 50)**			
50–59	1.0	0.5	2.1
60+	1.0	0.4	2.1
**Family medicine**	0.5	0.3	0.9
**Northeast**	1.2	0.6	2.3
**Urban setting**	1.4	0.8	2.6
**Solo, private practice**	0.7	0.3	1.5
**Intervention**	1.3	0.4	3.6
**Round**	1.9	0.7	5.0
Intervention * round	4.1	1.1	14.4
**Constant**	0.2	0.1	0.4

**Table:** 

(c)			
		[95% Conf. Interval]	
Variable	Odds Ratio	Lower	Upper
**Male**	0.5	0.2	1.1
**Age group (ref age < 50)**			
50–59	0.9	0.3	2.2
60+	0.9	0.3	2.5
**Family medicine**	0.8	0.4	1.8
**Northeast**	0.5	0.2	1.2
**Urban setting**	1.2	0.5	2.9
**Solo, private practice**	1.5	0.6	3.8
**Diagnosis of nutritional deficiency**	61.2	24.0	156.1
**Intervention**	3.2	0.8	12.8
**Round**	1.6	0.4	6.4
Intervention * round	6.3	1.2	33.6
**Constant**	0.0	0.0	0.2

**Table:** 

(d)			
		[95% Conf. Interval]	
Variable	Odds Ratio	Lower	Upper
**Male**	1.2	0.8	1.9
**Age group (ref age < 50)**			
50–59	0.9	0.6	1.5
60+	0.8	0.5	1.3
**Family medicine**	0.8	0.6	1.2
**Northeast**	0.9	0.6	1.5
**Urban setting**	1.1	0.7	1.7
**Solo, private practice**	1.1	0.7	1.8
**Intervention**	0.8	0.5	1.4
**Round**	0.7	0.4	1.2
Intervention * round	1.8	0.8	3.7
**Constant**	0.6	0.3	1.1

* Linear regression model; † logistic regression model.

## Supplementary Materials

The following are available online at https://www.mdpi.com/2075-4418/10/8/513/s1, Table S1: Biomarkers Known to Play a Role in Chronic Pain.
