# Supplementary material for: Randomized Trial on the Clinical Utility of a Novel Biomarker Panel to Identify Treatable Determinants of Chronic Pain
Source: Diagnostics (Basel). 2020 Jul 23;10(8):513. doi: 10.3390/diagnostics10080513 (PMC7459523; doi:10.3390/diagnostics10080513)
Supplement: Supplementary file 1 [file diagnostics-10-00513-s001.pdf]

Table 1. Biomarkers Known to Play a Role in Chronic Pain Tested by FBP.

Biomarker	Clinical Interpretation	Relevance to Pain and Clinical Features	Notes	Recommended Intervention
Methylmalonic acid	Elevated levels indicate a Vitamin B12 deficiency	Vitamin B12 deficiency leads to nerve damage and degeneration of the spinal cord. Peripheral neuropathy is the most common pain presentation	Deficiency can be precipitated by:<br>- Medications which lower stomach pH (e.g., proton pump inhibitors, H2 blockers)<br>- Metformin<br>- Gastric surgery or resection<br>- Vegan or vegetarian diets<br>- Exposure to nitrous oxide	Methylcobalamin 1-2 mg daily PO
Xanthurenic acid	Elevated levels indicate a Vitamin B6 deficiency	Neuropathy to due to Vitamin B6 deficiency starts with numbness, paraesthesias, or burning pain in the feet which then ascends to affect the legs and hands	Vitamin B6 deficiency can be caused by:<br>- Use of Vitamin B6 antagonists (INH, phenelzine, hydralazine, penicillamine, carbidopa)<br>- Hemodialysis<br>- Inflammatory or autoimmune disease	Vitamin B6 (pyridoxal 5 phosphate) 40 mg daily. Titrate accordingly.
Homocysteine	Elevated levels commonly indicate a B-Vitamin (B6/ B9/B12) deficiency.	Elevated homocysteine results from B-Vitamin deficiencies. Elevated homocysteine levels cause inflammation by increasing arachidonic acid and the proinflammatory prostaglandin E2 production.	Elevated homocysteine levels result from:<br>- B-Vitamin (B6/B9/B12) deficiencies<br>- Use of diuretic medications<br>- Chronic alcohol consumption	1) Address B6 or B12 deficiencies first as these are the most common cause for elevated homocysteine<br>2) If no B Vitamin deficiencies are identified then supplement with TMG (trimethylglycine) 400mg-600mg daily
3-HPMA	Elevated levels indicate increased exposure to acrolein	Acrolein contributes to inflammatory pain sensitivities through its binding and activation of the transient receptor potential ankyrin 1 receptor (TRPA1) in nerve fibers.	Increased acrolein exposure can result from:<br>- Chronic tobacco use<br>- Foods cooked or fried at very high temperatures<br>- Use of certain anti-cancer drugs<br>- Spinal cord injury	1) Prioritize N-acetyl-L-cysteine 600mg-2000 mg daily<br>2) Alpha Lipoic Acid 500-2000mg daily<br>(Note: Minimizing consumption of foods that contain added sweeteners and foods cooked at high temperatures can help inhibit the release of acrolein, thus reducing 3-HPMA levels.)
Pyroglutamate	Elevated levels indicate glutathione depletion	Glutathione depletion renders nerve cells susceptible to oxidative damage which can lead to neuropathic pain	Glutathione depletion can be caused by:<br>- Chronic use of Acetaminophen<br>- Poorly controlled Diabetes	1) N-acetyl-L-cysteine 600-1200mg<br>2) Alpha lipoic acid 600-1200mg daily

Ethylmalonate	Elevated levels indicate a carnitine deficiency	Carnitine deficiencies cause muscle aches and fatigue	Carnitine deficiency can be precipitated by:<br>- Valproic acid<br>- High fat diets<br>Treatment with Acetyl-L-Carnitine has been shown to improve nerve conduction and neuropathic pain symptoms	1) Acetyl-L-Carnitine 500-2000 mg daily<br>2) Phosphatidyl-choline 200mg-2g daily
Hydroxymethyl-glutarate	Elevated levels indicate a Coenzyme Q10 deficiency	Coenzyme Q10 deficiencies can cause muscle weakness and pain	Coenzyme Q10 deficiency can be precipitated by use of statin medications<br><br>Coenzyme Q10 supplementation ameliorates statin-associated muscle symptoms such as muscle pain and weakness	Co-enzyme Q10 50-250mg daily
5-HIAA	Abnormally low levels indicate decreased synthesis/turnover of serotonin	Abnormally low synthesis/turnover of serotonin can heighten pain sensitivity	Abnormally low synthesis/turnover of serotonin can be precipitated by:<br>- Chronic inflammation upregulates indoleamine 2,3-dioxygenase which redirects dietary tryptophan down the Kynurenine pathway and away from serotonin synthesis<br>- Insufficient intake of high-quality protein<br>- Vitamin B6 deficiency	1) 5-HTP 100-200 mg daily<br>2) Vitamin B6 (pyridoxal 5 phosphate) 40mg daily.
Kynurenate	Elevated levels indicate cytokine-mediated chronic inflammation	Kynurenate is a neuroactive Kynurenine Pathway (KP) metabolites which serves as a sensitive marker of chronic, systemic inflammation.<br>Upregulation of this pathway has been shown to play a central role in the comorbidity of pain and depression.	Chronic, systemic inflammation can be precipitated by:<br>- Autoimmune disease<br>- Exposure to LPS from gram negative bacteria	**If xanthurenate also elevated**: Vitamin B6 (pyridoxal 5 phosphate) 40mg daily<br>**If quinolinate also elevated**:<br>1) Magnesium glycinate 200-500mg daily<br>2) Nicotinamide 500-2000mg daily

Note: Reprinted from Clinical Validation of a Multi-Biomarker Assay for the Evaluation of Chronic Pain Patients in a Cross-Sectional, Observational Study in Pain and Therapy by Amirdelfan et al. Copyright 2020 by Springer Nature Switzerland AG.
